# A review of European studies on pollination networks and pollen limitation, and a case study designed to fill in a gap

**DOI:** 10.1093/aobpla/ply068

**Published:** 2018-10-31

**Authors:** Joanne M Bennett, Amibeth Thompson, Irina Goia, Reinart Feldmann, Valentin Ştefan, Ana Bogdan, Demetra Rakosy, Mirela Beloiu, Inge-Beatrice Biro, Simon Bluemel, Milena Filip, Anna-Maria Madaj, Alina Martin, Sarah Passonneau, Denisa P Kalisch, Gwydion Scherer, Tiffany M Knight

**Affiliations:** 1 Institute of Biology, Martin Luther University Halle-Wittenberg, Am Kirchtor, Halle (Saale), Germany; 2 German Centre for Integrative Biodiversity Research (iDiv), Halle-Jena-Leipzig, Deutscher Platz, Leipzig, Germany; 3 Department Community Ecology, Helmholtz Centre for Environmental Research – UFZ, Theodor-Lieser-Straße, Halle (Saale), Germany; 4 Faculty of Biology and Geology, Babeș-Bolyai University, Cluj-Napoca, Romania; 5 Helmholtz Centre for Environmental Research – UFZ, Permoserstr. 15, Leipzig, Germany; 6 Department for Integrative Zoology, Faculty of Life Sciences, University Vienna, Althanstrasse, Vienna, Austria; 7 University of Bayreuth, Department of Biogeography, Bayreuth, Germany; 8 University of Freiburg, Chair of Environmental Governance, Freiburg, Germany; 9 Faculty of Biology and Biotechnology, Ruhr University Bochum, Universitätsstraße, Bochum, Germany; 10 Nees Institut für Biodiversität der Pflanzen, Rheinische Friedrich-Wilhelms-Universität Bonn, Bonn, Germany; 11 Osnabrück University, Department of Biodiversity and Landscape Ecology, Barbarastraße, Osnabrück, Germany

**Keywords:** Meadows, monitoring, plant–pollinator interactions, plant–pollinator, networks, pollen limitation; butterfly

## Abstract

Anthropogenic environmental change disrupts interactions between plants and their animal pollinators. To assess the importance of different drivers, baseline information is needed on interaction networks and plant reproductive success around the world. We conducted a systematic literature review to determine the state of our knowledge on plant–pollinator interactions and the ecosystem services they provide for European ecosystems. We focussed on studies that published information on plant–pollinator networks, as a community-level assessment of plant–pollinator interactions and pollen limitation, which assesses the degree to which plant reproduction is limited by pollinator services. We found that the majority of our knowledge comes from Western Europe, and thus there is a need for baseline assessments in the traditional landscapes of Eastern Europe. To address this data gap, we quantified plant–pollinator interactions and conducted breeding system and pollen supplementation experiments in a traditionally managed mountain meadow in the Western Romanian Carpathians. We found the Romanian meadow to be highly diverse, with a healthy plant–pollinator network. Despite the presence of many pollinator-dependent plant species, there was no evidence of pollen limitation. Our study is the first to provide baseline information for a healthy meadow at the community level on both plant–pollinator interactions and their relationship with ecosystem function (e.g. plant reproduction) in an Eastern European country. Alongside the baseline data, we also provide recommendations for future research, and the methodological information needed for the continued monitoring and management of Eastern European meadows.

## Teaser

Our goal was to identify and bring awareness to research gaps in European pollination ecology. By systematically searching literature on plant–pollinator networks and pollen limitation we found a strong Western European bias in the location of studies and no baseline assessment of plant–pollinator interaction in Eastern European landscapes. To address this data gap, we quantified plant–pollinator interactions and conducted breeding system and pollen supplementation experiments in a traditionally managed meadow in the Eastern Romanian Carpathians. For future research, we provide the methodological information to continue monitoring and managing the meadows of Eastern Europe.

## Introduction

Europe has a long history of human use of grassland ecosystems as hay meadows; these ecosystems currently contain high biodiversity, being one of only two global community types that contain a global plant species richness maximum (Wilson *et al.* 2012) and are thus considered a global conservation priority (Habel *et al.* 2013). Recent changes in agricultural practices over the past few decades, such as agricultural intensification and abandonment, threaten these species-rich meadows (Strohbach *et al.* 2015). In Western Europe, the decline of traditional farming practices has meant many of these meadows have already been lost and those that are left are heavily managed for conservation purposes at considerable cost (Milberg *et al.* 2017). Eastern European countries still maintain high cover of traditionally managed landscapes and conserving these traditional landscapes is considered an important conservation priority (Fischer *et al.* 2012). However, there are biodiversity data gaps in Eastern Europe (Boakes *et al.* 2010) due to the ex-soviet governments’ tight controls on science funding and the low priority that was placed on conservation research (Pain and Travis 2009). Since joining the European Union, rapid economic development has led to an increasing rate of land-use abandonment and change in Eastern European countries, providing an imperative to collect baseline data in these areas (Culbert *et al.* 2017).

Many baseline conservation assessments include listings of plant and pollinator species diversity in a given locality. However, additional information that characterizes these interactions, such as the structure and stability of plant–pollinator interaction networks (Bascompte and Jordano 2007), the reliance of wild plants on pollination for reproduction (Aguilar *et al.* 2006), and the magnitude of pollen limitation for wild plants (Knight *et al.* 2005), provides a richer understanding of the structure of communities and the consequences that anthropogenic changes might have.

Plant–pollinator networks provide a community-level description of the presence of interactions between each species of plant and pollinator in a community. Mutualistic networks are typically found to be nested, in which specialist species tend to interact with generalist partners (Bascompte and Jordano 2007). Such a structure allows high stability of the network, since perturbations that cause extinctions of specialists will not typically result in cascading extinctions in the community (Memmott *et al.* 2007; Kaiser-Bunbury *et al.* 2010). Network analyses can provide information on the structure of these interactions, such as the degrees of connectedness and nestedness, as well as pinpoint key species, whose loss would have the potential to cause cascading extinctions in the community (Memmott *et al.* 2007; Kaiser-Bunbury *et al.* 2010).

Pollen supplementation experiments allow a quantitative assessment of the degree to which the reproduction of plant species is limited by the services of animal pollinators (Knight *et al.* 2005). The magnitude by which experimental pollen additions result in higher reproductive success in plants (i.e. the magnitude of pollen limitation) will depend on plant breeding systems (Knight *et al.* 2005). For example, pollinator-independent plants can avoid pollen limitation compared with plants that are dependent on pollinators for reproduction. Anthropogenic changes to the environment are known to cause pollen limitation (Knight *et al.* 2005).

The first goal of this study was to quantify the distribution of data on plant–pollinator networks and pollen limitation for wild plants in Europe, and to determine if there are data gaps for Eastern Europe. To achieve this aim, we conducted a systematic literature search for published plant–pollinator network studies and utilized an exhaustive global database of pollen limitation experiments (Bennett *et al.* 2018) to assess the state of our knowledge in Europe. We found that most of our knowledge comes primarily from Western Europe, and there is a need for these baseline assessments on plant–pollinator interactions and pollen limitation in less disturbed Eastern European landscapes. Thus our second goal was to describe a plant and pollinator community, in a single, highly diverse and traditionally managed meadow in Romania to rigorously document plant–pollinator interactions and pollen limitation, which provide a first step in filling in regional gaps in our knowledge.

## Methods

### Literature review

To obtain data on the distribution of plant–pollinator network studies, we conducted a systematic literature search using ISI’s Web of Science Core Collection. We started with a global distribution, as identifying information about whether the study was conducted within Europe is not always available in the title, abstract or keywords. The search terms used in the literature search were ‘pollination*’, ‘pollinator*’, ‘network*’, ‘interact*’ and ‘plant*’ in a variety of different word combinations **[see****Supporting Information S1****]**. We found 1054 total studies published between 1993 and February 2018. We divided these studies between four people, and each person read the abstracts (and subsequent sections of each manuscript, when the abstract looked promising) to identify studies that provided empirical data on plant–pollinator interactions at the community level. We included studies that focussed on multiple plant species and at least one Order of pollinating insects, typically presented as a network. One hundred and eighty-six studies were identified that met these criteria. These identified studies were then checked by a second person to verify their compliance with our inclusion criteria. Of these studies, 65 were conducted on the continent of Europe. Each study that met our criteria was inputted into a database documenting location (i.e. the country in which the study was conducted).

Data on the European distribution of pollen limitation experiments were extracted from GloPL, a global dataset on pollen supplementation experiments designed to quantify the magnitude of pollen limitation for wild plant species (Bennett *et al.* 2018). To determine the number of pollen limitation studies per country, we spatially intersected the coordinates in the GloPL database of pollen limitation studies provided by Bennett *et al.* (2018) with the GADM, database of global administrative areas (as shapefile—downloaded from http://www.gadm.org/). For studies without coordinates, we used the location description column to determine the country of study. From the 927 unique studies in the GloPL dataset, 143 were conducted on the continent of Europe. We then visualized the number of plant–pollinator network studies and pollen limitation studies per country using a thermic map using the tmap package (Tennekes 2018) in R (R Core Team 2017).

### Romanian field study

#### Study site

Romania’s traditional low intensity agricultural practices and high biogeographical and habitat diversity has translated into a remarkably high species diversity, with approximately 228 endemic and subendemic species (Ioras 2003). Our study was carried out in a mountain meadow in Ghețari village (46°29′N; 22°49′E). Ghețari village is located in the upper basin of the Arieş River, Apuseni Mountains (Western Romanian Carpathians, Apuseni Natural Park) and lies on the Ocoale-Scărișoara carstic plateau. The region has a mountain climate, with a proximate mean annual temperature of 4.5^o^C and mean annual precipitation of 1145.26 mm (Rușdea *et al.* 2005). The dominant soils are eutrophic and mesotrophic brown soils, brown rendzina soils, rendzins, terra rossa, rarely brown acid, gleic and podzolic soils (Rușdea *et al.* 2005). The mountain forests are predominantly spruce, beech and mixed forests. The meadows belong to *Molinio-Arrenatheretea* and *Festuco-Brometea* classes, and are edified by *Festuca rubra*, *Trisetum flavescens*, *Cynosurus cristatus*. Our focal meadow is traditionally managed. It is mowed once per year and manure is applied during late autumn or winter. During autumn, the meadow is occasionally grazed (no more than one cow/ha). The meadow was 3.8 ha and bordered on two sides by forest and on two sides by a hedgerow that separated our focal meadow from another meadow.

#### Survey of plant diversity

We surveyed the diversity of plants in bloom from 10 to 14 July 2017. The field team walked through the meadow, searched for blooming plants, and identified these to species. In total, 86 plant species were blooming during our study period. Plants were placed in abundance categories (high, medium and low) based on the abundance of blooming individuals. A plant species can be high in abundance based on vegetative cover, but not categorized in our high category if it is past its peak flowering time. The abundance categories were high (>50 blooming individuals), medium (20–50 blooming individuals), low (<20 blooming individuals). Thirty-three species were placed in the high-abundance category. All of these were considered in our plant–visitor network study and a subset was selected for pollen supplementation and breeding system experiments. Appendix 1 provides a list of all plant species in bloom and their abundance category.

#### Survey of butterfly diversity

Butterflies are commonly used for monitoring and to set conservation priorities in Europe (Van Swaay and Warren 1999). Thus, we listed all of the butterfly species at the field site. An expert walked the field site from 10 to 14 July 2017, netting individuals and identifying them to species. Based on the trapping intensity of each species, we estimated the abundance of each species on the meadow. Appendix 2 is a list of all butterfly species with categorized abundance (very rare (1–2 individuals observed), rare (3–9 individuals observed), common (10–50 individuals observed) and very common (50–1000 individuals observed)).

#### Plant–pollinator network

We spent approximately 60 person hours (15 persons) collecting plant–pollinator network data from 10 to 14 July 2017. We focussed on 33 flowering species in the ‘abundant’ category. For each plant species, we recorded the number and identity of visiting insects and used sweep nets to collect insect visitors that were unable to be identified in the field. Collections occurred on all days for several hours between 10.30 and 16.30. Insects that could be identified in the field (*Apis mellifera*, many *Bombus* spp., Lepidoptera) were recorded and released. Other insects were collected in vials and labelled with the plant species it was collected from, the date of collection, and the initials of the collector. Duplicates of collected insects were counted, recorded and released. The insects were frozen, pinned, and later identified using published taxonomic guides (Oosterbroek 2006; Bartsch 2009; van Veen 2009; Chinery 2012) and assistance from experts. Hymenoptera, Diptera and Lepidoptera known to pollinate were included in network analyses, while other insects that are not considered pollinators were excluded (Orthoptera, Hemiptera, Formicidae, Coleoptera). Coleoptera were not included in the network because of the difficulty of distinguishing between species that are pollen-facilitators and those that are pollen eaters.

For each of the focal flowering plant species in our network, we sampled insect individuals until we reached saturation on species richness observed as a function of insect individuals observed. We used a Chao species richness estimator (Colwell and Coddington 1994) to extrapolate ‘true’ insect richness for each of our plant species based on our abundance data.

#### Compatibility system and pollen supplementation experiments

To quantify the degree to which plants relied on animal pollinators, and the degree to which their reproductive fitness was limited by pollen receipt, we conducted breeding system and pollen supplementation experiments on nine-focal abundant plant species. Plant species in high-abundance category that were in the beginning phases of flowering, meaning most flowers were un-opened buds were chosen for the experiments. These species were *Campanula serrata*, *Cirsium erisithales*, *Dianthus carthusianorum*, *Helianthemum nummularium*, *Hypericum perforatum*, *Lotus corniculatus*, *Scabiosa columbaria*, *Sonchus arvensis* and *Trollius europaeus*.

To conduct the experiments, we randomly assigned flowers, inflorescences or plants at a similar phenological stage and size to one of the three treatments: control, bagged and supplement. Each treatment had approximately 10 replicates per species. In the control treatment, all flowers were open-pollinated and unmanipulated. Flowers, inflorescences or plants in the bagged treatment were covered with a mesh bag (lightweight ‘bridal veil’) before opening to exclude pollinators, thus any reproduction in this treatment is due to autogamous selfing. Supplemented flowers were open-pollinated flowers that received additional pollen collected from one to three donor plants of the same species located at least 10 m away from the focal plant. Pollen was brushed on or inserted using tweezers until saturation. Reproductive success was quantified as seeds per fruit or flower (*C. serrata, C. erisithales, L. corniculatus, S. solumbaria, T. europaeus*) or seeds per plant (*D. carthusianorum, H. nummularium, H. perforatum, S. arvensis*).

The degree of autogamy for each species is calculated as the ratio of mean reproductive success in the bagged versus the supplement treatment. Where a value ≥0.2 indicates a species is self-incompatible and dependent on pollinators for its reproduction, and a value ≤0.8 indicates that the species is not pollinator dependant (Schoen and Lloyd 1992; Rodger and Ellis 2016). The magnitude of pollen limitation is calculated as the effect size (ES) between the supplement and control treatment using the log response ratio where ES = ln(reproductive success in supplement treatment) – ln(reproductive success in control treatment). A species is considered pollen limited when pollen supplemented flowers have a higher reproductive output (i.e. seed or fruit production) relative to naturally pollinated flowers (i.e. a positive effect size) (Knight *et al.* 2005).

### Statistical analyses

We described the structure of the plant–pollinator network using two metrics, nestedness and connectivity, which have been linked to network stability in simulation studies that manipulate the cascading effects of species loss on secondary extinctions (Fortuna and Bascompte 2006). We calculated nestedness using the bipartite package in R (Dormann *et al*. 2008). For the breeding system and pollen supplementation experiments, we used a one-way ANOVA followed by Tukey Tests to test if there were significant differences in reproduction among treatments. All statistical operations were performed in R (R Core Team 2017).

## Results

**Figure 1.**
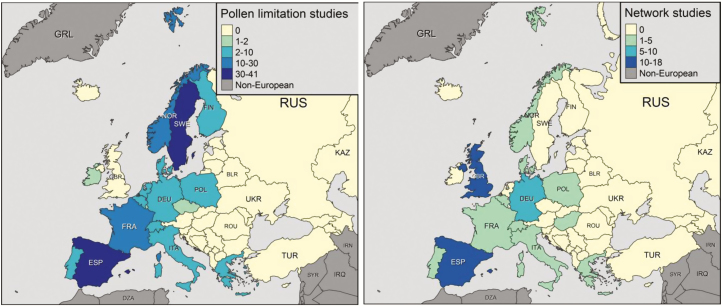
Locations of studies conducted in Europe on (A) pollen limitation and (B) plant–pollinator networks.

We find that the large majority of published studies investigating pollen limitation or plant–pollinator networks are conducted in Western Europe (Fig. 1). For example, our study is the first documenting the magnitude of pollen limitation and the structure of plant–pollinator networks in Romania. With the exception of Poland, no other Eastern European country has data on both pollen limitation and plant–pollinator networks.

The data we collected at one species-rich meadow shows that there is a high amount of diversity of both plants and pollinators. There were 86 species of plants that were flowering during our week of sampling in the meadow **[see****Supporting Information****—****Table S2****]**, which included *Arnica montana* and *C. serrata*, which are species considered to be a conservation interest under the Habitats Directive (1992) and 23 species known to be important for medicinal purposes and/or used as a food resource (IUCN 2015). A total of 38 butterfly species from seven families were detected in this single meadow. While this is an impressive number, it represents <19 % of the known day butterfly fauna from Romania (203 estimated species) (Rákosy *et al.* 2003). Of the species observed, 13 species are considered very rare (34 %), 10 species rare (26 %), 8 species common (21 %) and 7 species very common (18 %) **[see****Supporting Information—Table S3****]**.

**Figure 2.**
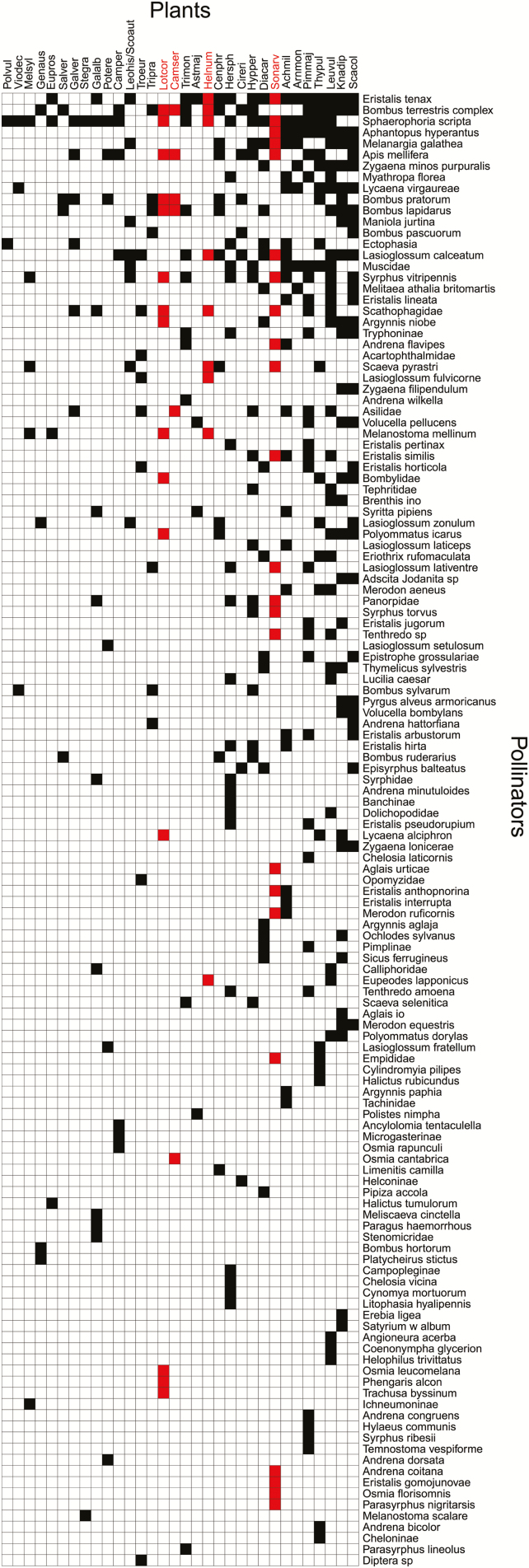
Plant–pollinator network of 33 flowering plant species and 132 pollinator species. Pollinators and plants are in rank order according to their number of links. Filled boxes indicate interactions observed between a plant and pollinator species (1911 total links). Four focal plant species for the breeding system and pollen supplementation experiments that were pollinator dependent are highlighted in red.

The plant–pollinator network consisted of 132 unique pollinators species from three orders (Diptera, Hymenoptera and Lepidopera) collected on 33 plant species with 1911 links. There were 63 Dipteran species, 42 Hymenopteran species and 27 Lepidopteran species (Fig. 2). The temperature of the network was 8.54, indicating that the network was relatively well nested.

Only two of our nine-focal species showed high levels of autogamy, while four species were significantly pollinator dependent (Table 1). Despite their high reliance on animal pollinators for reproduction, none of our nine-focal species were significantly pollen limited. The four pollinator-dependent species were well connected within the network (Fig. 2).

**Table 1.** Results for nine-focal plant species including mean, standard error (SE), and sample size (N) for the three treatments: supplement (S), control (C), and bagged (B), used in the compatibility system and pollen supplementation experiment. Viable seeds were counted either as per flower, plant, or by fruit. Species are ordered alphabetically. All plants are perennial herbs. The adjusted *P*-values for Tukey tests are given for each treatment. Two plant species were significantly pollinator dependent and two marginally significant (Tukey S-B). No species were pollen limited (Tukey S-C). Results for the network connectance are given: the number of links for each plant species (number of visiting species), the estimated number of links (Chao estimator), and the total number of visitors observed. *Results are marginally significant. **Results are significant.

Species		S mean ± SE (N)	C mean ± SE (N)	B mean ± SE (N)	Tukey (S-B) *P*-value	Tukey (C-B) *P*-value	Tukey (S-C) *P*-value	No. of links (visiting species)	Chao estimator (SE)	No. of visitors (individuals)
*Campanula serrata*	Seeds per flower	70.83 ± 39.33 (6)	76 ± 19.13 (8)	0 ± 0 (10)	0.042**	0.0021**	0.60	6	9 (4.1)	35
*Cirsium erisithales*	Seeds per flower	41.75 ± 11.39 (8)	47.88 ± 9.44 (8)	12.2 ± 3.57 (5)	0.19	0.12	0.94	7	10 (4.44)	75
*Dianthus carthusianorum*	Seeds per plant	37.5 ± 10.73 (8)	12.75 ± 4.35 (8)	14.83 ± 5.06 (6)	0.94	0.55	0.73	19	28 (7.6)	84
*Helianthemum nummularium*	Seeds per plant	13.5 ± 3.96 (10)	16.5 ± 4.03 (10)	4.1 ± 2.08 (10)	0.069*	0.21	0.82	9	10.5 (2.56)	51
*Hypericum perforatum*	Seeds per plant	153.5 ± 42.37 (10)	228.33 ± 65.32 (6)	24.62 ± 10.11 (8)	0.16	0.15	0.96	18	36 (16.06)	76
*Lotus corniculatus*	Seeds per fruit	2.67 ± 1.36 (6)	14.9 ± 5.26 (10)	0 ± 0 (9)	0.061*	0.0016**	0.53	15	30 (12.8)	34
*Scabiosa columbaria*	Seeds per flower	45.44 ± 8.75 (9)	56 ± 10.19 (10)	41.67 ± 6.68 (9)	0.43	0.99	0.52	31	46.6 (11.63)	346
*Sonchus arvensis*	Seeds per plant	68.5 ± 19.06 (10)	110.4 ± 24.86 (10)	16.4 ± 6.53 (10)	0.00017**	0.0000016**	0.20	23	38 (10.33)	89
*Trollius europaenus*	Seeds per flower	165 ± 41.15 (3)	82.38 ± 21.17 (8)	0 ± 0 (8)	0	0	0.99	8	11 (4.11)	32

## Discussion

Many Eastern European countries still have vast landscapes of hay meadows that are managed traditionally and have not been strongly affected by industrial agricultural practices (Rușdea *et al.* 2005; Konvicka *et al.* 2006). At small spatial scales, these meadows are some of richest places for biodiversity in Europe (e.g. Wilson *et al.* 2012). As these countries continue to develop, the quality of the ecosystems could decline. It is, therefore, critical that the diversity and interactions of these ecosystems are thoroughly documented, so that we can understand patterns within single sites, as well as how these patterns vary across space and time. Such baseline information will allow us to set priorities for the conservation of plants and pollinators. Further, baseline information will allow us to monitor and detect how climate and land-use change alter the diversity, composition and structure of plants, insects and their interactions within meadows. Despite the urgent conservation need, we find that the current baseline information available for understanding the structure of plant–pollinator interactions and the degree to which pollination currently limited plant reproductive success are completely absent from the scientific literature in many Eastern European countries.

Our knowledge of plant–pollinator interactions at the community level in Eastern Europe comes from four published studies and from the empirical research in Romania that we present in this manuscript. Of the published studies, one is in a forest ecosystem (Albrecht *et al.* 2014), one is in an urban environment (Jędrzejewska-Szmek and Zych 2013) and two are in meadows. Of the two studies in Eastern European meadows (Goldstein and Zych 2016; Szigeti *et al.* 2016), the methods are quite different from ours (e.g. one considers interactions in small plots, the other does not present their interaction as a network), and thus direct comparison with our results is not possible. Moving forward, studies that consider similar sampling methodology are necessary to provide the robust baseline data we need for conservation.

In our Romanian meadow, we found an interaction network that has features that are typical of a healthy mutualism network, such as a nested structure and more pollinator species than plant species (Memmott *et al.* 2004). The network also has a diverse plant and pollinator community, although the observed number of insect species was about a third lower than the diversity suggest by the Chao estimator (Table 1), but is similar to results found by Forup and Memmott (2005) when they looked at restored hay meadows in Great Britain. Butterflies are one of the very few taxonomic groups and perhaps the only pollinating taxonomic groups for which Pan-European data are available (Van Swaay and Warren 1999). They are commonly used for monitoring and setting conservation priorities and are highly sensitive to global changes drivers, including climate and land-use change (Warren *et al.* 2001), making them ideal bio-indicators for across site comparisons. To facilitate future monitoring and provide the basis for broader comparisons between our site and other sites across Europe, we have provided a full species list of the butterfly species present at our site. However, no butterfly species were considered ‘hub’ species (i.e. most connected species) in our plant–pollinator network. Instead the three ‘hub’ pollinators in this network are two hoverflies (*Eristalis tenax* and *Sphaerophoria scripta*) and one bumble bee (*Bombus terrestris*). These are common and widely distributed pollinating species. Thus, there should be future opportunities to compare the connections that we find here with those found in other European plant–pollinator networks. The ‘hub’ plant species are *Scabiosa columbaria*, *Knautia dipsacifolia* and *Leucanthemum vulgare*. These are also species found in many other European countries and should allow for broad comparisons. For example, one study in Great Britain found that *Leucanthemum vulgare*, also a very common species in their study fields, was highly visited by a group of plant-visitors (Dicks *et al.* 2002). However, some of our plant species are typical of nutrient-poor meadows and might be rare or absent in meadows that have more anthropogenic influences, for example, *A. montana*, *C. serrata* and some orchids.

There were three published pollen supplementation experiments from Eastern Europe, all conducted in grasslands. Similar to our study, the two Polish studies were in hay meadows and both found their focal species to be pollinator dependent (Zych and Stpiczyńska 2012; Zych *et al.* 2013). Specifically, Zych and Stpiczyńska (2012) found *Fritillaria meleagris* to be self-compatible but not autofertile or pollen limited. Zych *et al.* (2013) found the red listed species *Polemonium caeruleum* to be self-incompatible and at risk of pollen limitation in years of low pollinator abundance. The single study from the Czech Republic was conducted on a different grassland habitat type, rocky steppe, and found their focal species *Dracocephalum austriacum* to be pollen limited despite being moderately autofertile (Castro *et al.* 2015). Noticeably, all prior work in Eastern Europe documented the breeding system and level of pollen limitation on a single focal species. This makes, our multi species study the first to be able to draw inferences about plant reproductive strategy and reproductive success at the community level. Only one of our focal species, *Lotus corniculatus,* was previously studied in Norway and was found in GloPL. Similar to our study, no evidence of pollen limitation was detected and even had higher seed production in the control treatment (Hegland and Totland 2008). In the future, if we are to understand the effects of global change, including land-use abandonment and intensification on these rapidly changing meadows and make informed conservation management decisions, more community-level studies are needed. Many of the species in our study are pollinator dependent and thus susceptible to changes in pollinator communities. These species are found across Europe, and thus our study could provide the bases for a broader comparison between sites under differing levels and types of anthropogenic disturbance in different countries across Europe.

## Conclusion

As Eastern Europe continues to develop, traditional farming practices are declining, putting traditional meadows and the ecosystems services they provide at risk. Our study provides the first data on multispecies plant–pollinator interactions from Eastern Europe. In addition, we offer a framework for further studies by assessing potential target plant species for the continued monitoring of meadow ecosystem function in relation to pollination services. Thus, our study could set the foundations for collecting data that would inform conservation priorities for plants and pollinators in these rapidly changing systems.

## Data

Codes written for results are available at GitHub at https://github.com/idiv-biodiversity/plant-pollinator-romania. It includes code to replicate the maps in Fig. 1 (Work flow for maps) which uses data found in **Supporting Information S4** and to plot the network matrix in Fig. 2 (Workflow for the web plot) which uses data found in **Supporting Information S5**.

## Supporting Information

The following supporting information is available in the online version of this article:

**Text S1**. Pollination Network Literature Search.

**Table S2**. A list of all plant species recorded flowering in the meadow, if they were considered highly abundant (over 50 blooming individuals), included in the pollen limitation and breeding experiments, whether they are priorities for conservation and included in the habitat directive (Habitats Directive 1992) and their societal use (IUCN 2015).

**Table S3**. A list of all butterfly species observed in the meadow.

**Table S4**. The number of published studies conducted in Europe containing a plant–pollinator network illustrated in Fig. 1.

**Table S5**. Data for the plant-pollinator network matrix presented in Fig. 2.

Supplementary Information S1

Supplementary Table S2

Supplementary Information S3

Supplementary Information S4

Supplementary Information S5

## Sources of Funding

Funding was provided by the Alexander von Humboldt Foundation as part of the Alexander von Humboldt Professorship of T.M.K and by the Helmholtz Association as part of the Helmholtz Recruitment Initiative. Further support for the literature review on pollen supplementation experiments was provided by sDiv, the synthesis centre of iDiv (German Centre for Integrative Biodiversity Research).

## Contributions by the Authors

J.M. B. and A.T. lead field data collection, the literature search, data analysis and the writing of the manuscript. I.G. and R.F. lead field data collection and contributed to the writing of the manuscript. V.Ş. contributed towards field data collection, lead analysis in the manuscript and commented on drafts of the manuscript. A.B., D.R., M.B., I-B.B., S.B., M.F., A-M.M. and A.M. contributed towards field data collection and commented on drafts of the manuscript. S.P. lead the literature search and contributed to the writing of the manuscript. D.P.K. contributed towards field data collection and commented on drafts of the manuscript. G.S. contributed towards field data collection and commented on drafts of the manuscript. T.M.K. conceived of the project, lead field data collection, the literature search and writing of the manuscript.

## Conflicts of Interest

None declared.

## References

[CIT0001] Aguilar R, Ashworth L, Galetto L, Aizen MA 2006 Plant reproductive susceptibility to habitat fragmentation: review and synthesis through a meta-analysis. Ecology Letters 9:968–980. 16913941 10.1111/j.1461-0248.2006.00927.x

[CIT0002] Albrecht J, Berens DG, Jaroszewicz B, Selva N, Brandl R, Farwig N 2014 Correlated loss of ecosystem services in coupled mutualistic networks. Nature Communications 5:3810. 10.1038/ncomms4810 24806612

[CIT0003] Bartsch H 2009 Tvåvingar: Blomflugor. Uppsala: ArtDatabanken.

[CIT0004] Bascompte J, Jordano P 2007 Plant-animal mutualistic networks: the architecture of biodiversity. Annual Review of Ecology, Evolution and Systematics 38: 567–5 93.

[CIT0005] Bennett JM, Steets JA, Burns JH, Durka W, Vamosi JC, Arceo-Gómez G, Burd M, Burkle LA, Ellis AG, Freitas L, Li J, Rodger JG, Wolowski M, Xia J, Ashman T-L, Knight TM 2018 GloPL, Global data base on pollen limitation of plant reproduction. Scientific Data 5:180249. 30457567 10.1038/sdata.2018.249 PMC6244188

[CIT0006] Boakes EH, McGowan PJ, Fuller RA, Chang-qing D, Clark NE, O’Connor K, Mace GM 2010 Distorted views of biodiversity: spatial and temporal bias in species occurrence data. Plos Biology 8:e1000385. 20532234 10.1371/journal.pbio.1000385 PMC2879389

[CIT0007] Castro S, Dostálek T, van der Meer S, Oostermeijer G, Münzbergová Z 2015 Does pollen limitation affect population growth of the endangered *Dracocephalum austriacum* L.?. Population Ecology 57(1):105–116.

[CIT0008] Chinery M 2012 Insects of Britain and Western Europe, 3rd edn. London, UK: A&C Black.

[CIT0009] Colwell RK, Coddington JA 1994 Estimating terrestrial biodiversity through extrapolation. Philosophical Transactions of the Royal Society of London. Series B, Biological Sciences 345:101–118. 7972351 10.1098/rstb.1994.0091

[CIT0010] Culbert PD, Dorresteijn I, Loos J, Clayton MK, Fischer J, Kuemmerle T 2017 Legacy effects of past land use on current biodiversity in a low-intensity farming landscape in Transylvania (Romania). Landscape Ecology 32:429–444.

[CIT0011] Dicks LV, Corbet SA, Pywell RF 2002 Compartmentalization in plant–insect flower visitor webs. Journal of Animal Ecology 71:32–43.

[CIT0012] Dormann CF, Gruber B, Fründ J 2008 Introducing the bipartite package: analysing ecological networks. Interaction 1:0.2413793.

[CIT0052] Fischer J, Hartel T, Kuemmerle T 2012 Conservation policy in traditional farming landscapes. Conservation Letters 5(3):167–175.

[CIT0013] Fortuna MA, Bascompte J 2006 Habitat loss and the structure of plant-animal mutualistic networks. Ecology Letters 9:281–286. 16958893 10.1111/j.1461-0248.2005.00868.x

[CIT0014] Forup ML, Memmott J 2005 The restoration of plant–pollinator interactions in hay meadows. Restoration Ecology 13:265–274.

[CIT0015] Goldstein J, Zych M 2016 What if we lose a hub? Experimental testing of pollination network resilience to removal of keystone floral resources. Arthropod–Plant Interactions 10:263–271.

[CIT0016] Habel JC, Dengler J, Janišová M, Török P, Wellstein C, Wiezik M 2013 European grassland ecosystems threatened hotspots of biodiversity. Biodiversity and Conservation 22:2131–2138.

[CIT0017] Habitats Directive 1992 Council Directive 92/43/EEC of 21 May 1992 on the conservation of natural habitats and of wild fauna and flora. Official Journal of the European Union 206:7–50.

[CIT0018] Hegland SJ, Totland Ø 2008 Is the magnitude of pollen limitation in a plant community affected by pollinator visitation and plant species specialisation levels? Oikos 117:883–891.

[CIT0019] Ioras F 2003 Trends in Romanian biodiversity conservation policy. Biodiversity and Conservation 12:9–23.

[CIT0020] IUCN 2018 The IUCN Red List of Threatened Species Version 2018-2. http://www.iucnredlist.org.

[CIT0021] Jędrzejewska-Szmek K, Zych M 2013 Flower-visitor and pollen transport networks in a large city: structure and properties. Arthropod–Plant Interactions 7:503–516.

[CIT0022] Kaiser-Bunbury CN, Muff S, Memmott J, Müller CB, Caflisch A 2010 The robustness of pollination networks to the loss of species and interactions: a quantitative approach incorporating pollinator behaviour. Ecology Letters 13:442–452. 20100244 10.1111/j.1461-0248.2009.01437.x

[CIT0023] Knight TM, Steets JA, Vamosi JC, Mazer SJ, Burd M, Campbell DR, Dudash MR, Johnston MO, Mitchell RJ, Ashman T-L 2005 Pollen limitation of plant reproduction: Pattern and Process. Annual Review of Ecology, Evolution, and Systematics 36:467–497.

[CIT0024] Konvicka M, Fric Z, Benes J Butterfly extinctions in European states: do socioeconomic conditions matter more than physical geography? Global Ecology and Biogeography 2006 15:82–92.

[CIT0025] Memmott J, Craze PG, Waser NM, Price MV 2007 Global warming and the disruption of plant-pollinator interactions. Ecology Letters 10:710–717. 17594426 10.1111/j.1461-0248.2007.01061.x

[CIT0026] Memmott J, Waser NM, Price MV 2004 Tolerance of pollination networks to species extinctions. Proceedings Biological Sciences 271:2605–2611. 15615687 10.1098/rspb.2004.2909 PMC1691904

[CIT0027] Milberg P, Tälle M, Fogelfors H, Westerberg L 2017 The biodiversity cost of reducing management intensity in species-rich grasslands: mowing annually vs. every third year. Basic and Applied Ecology 22:61–74.

[CIT0028] Oosterbroek P 2006 The European Families of Diptera. The Netherlands: KNNV.

[CIT0029] Pain E, Travis K 2009 After the fall of the wall: science careers in Eastern Europe. Science. doi: 10.1126/science.caredit.a0900135 http://www.sciencemag.org/careers/2009/11/after-fall-wall-science-careers-eastern-europe.

[CIT0030] Rákosy L, Goia M, Kovács Z 2003 Catalogul Lepidopterelor României/Verzeichnis Der Schmetterlinge Rumäniens. Cluj-Napoca: Societatea Lepidopterologica Română 446.

[CIT0031] R Core Team 2017 R: A language and environment for statistical computing. Vienna, Austria: R Foundation for Statistical Computing http://www.R-project.org/.

[CIT0032] Rodger JG, Ellis AG 2016 Distinct effects of pollinator dependence and self-incompatibility on pollen limitation in South African biodiversity hotspots. Biology Letters 12: 1–4. doi: 10.1098/rsbl.2016.0253. PMC4938053 27277954

[CIT0033] Rușdea E, Reif A, Povară I, Konold W 2005 Perspektiven für eine traditionelle Kulturlandschaft in Osteuropa. Culterra, Schriftenreihe des Instituts für Landespflege der Albert-Ludwigs-Universität Freiburg 34:1–449.

[CIT0034] Schoen DJ, Lloyd DG 1992 Self-and cross-fertilization in plants. III. Methods for studying modes and functional aspects of self-fertilization. International Journal of Plant Sciences 153:381–393.

[CIT0035] Strohbach MW, Kohler ML, Dauber J, Klimek S 2015 High nature value farming: from indication to conservation. Ecological Indicators 57:557–563.

[CIT0036] Szigeti V, Kőrösi Á, Harnos A, Nagy J, Kis J 2016 Comparing two methods for estimating floral resource availability for insect pollinators in semi-natural habitats. Annales de La Société Entomologique de France (NS). 52:289–299.

[CIT0037] Tennekes M 2018 tmap: thematic maps R package. tmap Themat Maps 1.

[CIT0038] Van Swaay CAM & Warren MS 1999 Red data book of European butterflies (Rhopalocera). Nature and Environment, No. 99. Strasbourg: Council of Europe Publishing.

[CIT0039] van Veen MP 2009 Hoverflies of Northwest Europe, 2nd edn. The Netherlands: KNNV.

[CIT0040] Warren MS, Hill JK, Thomas JA, Asher J, Fox R, Huntley B, Roy DB, Telfer MG, Jeffcoate S, Harding P, Jeffcoate G, Willis SG, Greatorex-Davies JN, Moss D, Thomas CD 2001 Rapid responses of British butterflies to opposing forces of climate and habitat change. Nature 414:65–69. 11689943 10.1038/35102054

[CIT0041] Wilson JB, Peet RK, Dengler J, Pärtel M 2012 Plant species richness: the world records. Journal of Vegetation Science 23:796–802.

[CIT0042] Zych M, Stpiczyńska M 2012 Neither protogynous nor obligatory out-crossed: pollination biology and breeding system of the European Red List *Fritillaria meleagris* L. (liliaceae). *Plant Biology* (*Stuttgart, Germany*) 14:285–294. 10.1111/j.1438-8677.2011.00510.x 21972995

[CIT0043] Zych M, Stpiczyńska M, Roguz K 2013 Reproductive biology of the Red List species *Polemonium caeruleum* (Polemoniaceae). Botanical Journal of the Linnean Society 173:92–107.

