# Supplementary material for: A review of European studies on pollination networks and pollen limitation, and a case study designed to fill in a gap
Source: AoB Plants. 2018 Oct 31;10(6):ply068. doi: 10.1093/aobpla/ply068 (PMC6302952; doi:10.1093/aobpla/ply068)
Supplement: Supplementary Information S1 [file ply068_suppl_supplementary_information_s1.pdf]

Supporting Information S1: Pollination Network Literature Search.

In January 2018 we used the five following search strings to ensure a complete coverage of published records of pollination network research were obtained 1) “pollination” and “network” and “interact”; 2) “pollination” and “network”; 3) “pollinator” and “network” and “interact”; 4) pollinator” and “network”; 5) pollinator” and “plant” and “network”. All records were downloaded and then collated into a single Excel file to remove duplicate records. Total number of unique records totaled: 1,054 studies. Each record was then reviewed to determine it’s compliance with inclusion criteria. Studies were not included if they did not have original plant-visitor network data or if they were not conducted at the community level i.e. when only one or a limited number of focal plant or insect species were studied.
