# Supplementary material for: A review of European studies on pollination networks and pollen limitation, and a case study designed to fill in a gap
Source: AoB Plants. 2018 Oct 31;10(6):ply068. doi: 10.1093/aobpla/ply068 (PMC6302952; doi:10.1093/aobpla/ply068)
Supplement: Supplementary Table S2 [file ply068_suppl_supplementary_table_s2.pdf]

Supporting Information Table S2: A list of all plant species recorded flowering in the meadow, if they were considered highly abundant (over 50 blooming individuals), included in the pollen limitation and breeding experiments, whether they are priorities for conservation and included in the habitat directive (Habitats Directive 1992) and their societal use (IUCN 2015).

Accepted species name (Species Code)	Abundant	Experiment	Habitat directive	Use
*Campanula serrata (*Camser*)*	y	y	Y	
*Cirsium erisithales (*Cireri*)*	y	y		
*Dianthus carthusianorum (*Diacar*)*	y	y		
*Helianthemum nummularium (*Helnum*)*	y	y		
*Hypericum perforatum(*Hypper*)*	y	y		medicinal
*Lotus corniculatus (*Lotcor*)*	y	y		
*Scabiosa columbaria (*Scacol*)*	y	y		
*Sonchus arvensis (*Sonarv*)*	y	y		
*Trollius europaeus (*Troeur*)*	y	y		
*Achillea millefolium (*Achmil*)*	y			medicinal, feed, food
*Arnica montana (*Arnmon*)*	y		Y	medicinal
*Astrantia major (*Astmaj*)*	y			medicinal
*Campanula persicifolia (*Camper*)*	y			
*Centaurea phrygia (*Cenphr*)*	y			
*Euphrasia rostkoviana (*Eurros*)*	y			medicinal
*Galium album (*Galalb*)*	y			
*Galium verum (*Galver*)*	y			food, medicinal
*Gentianella austriaca (*Genaus*)*	y			
*Heracleum sphondylium (*Hersph*)*	y			
*Knautia dipsacifolia (*Knadip*)*	y			
*Leontodon hispidus (*Leohis*)*	y			
*Leucanthemum vulgare (*Leuvul*)*	y			
*Melampyrum sylvaticum (*Melsyl*)*	y			
*Pimpinella major (*Pimmaj*)*	y			
*Polygala vulgaris (*Polvul*)*	y			medicinal
*Potentilla erecta (*Potere*)*	y			
*Salvia verticillata (*Salver*)*	y			
*Scorzoneroides autumnalis (*Scoaut*)*	y			
*Stellaria graminea (*Stegra*)*	y			
*Thymus pulegioides (*Thypul*)*	y			
*Trifolium pratense (*Tripra*)*	y			food
*Trifolium montanum (*Trimon*)*	y			

Viola declinata (Viodec)	y	
Aconitum vulparia (Acovul)		
Alchemilla monticola (Alcmon)		
Anacamptis coriophora (Anacor)		food
Antennaria dioica (Antdio)		medicinal
Anthyllis vulneraria (Antvul)		
Aspilia floribunda (Aspflo)		
Campanula patula subsp. Abietina (Campat)		
Campanula rapunculoides (Camrap)		
Carum carvi (Carcar)		food, medicinal
Clinopodium vulgare (Clivul)		medicinal
Dactylorhiza maculata (Dacmac)		food
Dactylorhiza majalis (Dacmaj)		food
Dianthus barbatus (Diabar)		
Digitalis grandiflora (Diggra)		medicinal
Doronicum austriacum (Doraus)		
Filipendula ulmaria (Filulm)		medicinal
Galeopsis ladanum (Gallad)		
Galium mollugo (Galmol)		
Gentiana cruciata (Gencru)		
Gnaphalium sylvaticum (Gnasyl)		
Gymnadenia conopsea (Gymcon)		
Jacobaea vulgaris (Jacvul)		
Laserpitium latifolium (Laslat)		
Lathyrus pratensis (Latpra)		
Linum catharticum (Lincat)		
Neottia ovata (Neoova)		
Medicago falcata (Medfal)		
Medicago lupulina (Medlup)		
Myosotis scorpioides (Myosco)		
Neotinea ustulata (Neoust)		food, medicinal
Origanum vulgare (Orivul)		medicinal
Parnassia palustris (Parpal)		medicinal
Pilosella aurantiaca (Pilaur)		
Pilosella officinarum (Piloff)		medicinal
Pimpinella saxifraga (Pimsax)		food
Podospermum roseum (Podros)		
Polygala nicaeensis (Polnic)		
Prunella vulgaris (Pruvul)		medicinal
Ranunculus acris (Ranacr)		
Rhinanthus minor (Rhimin)		
Rhinanthus rumelicus (Rhirum)		
Rumex acetosa (Rumace)		

Sanguisorba minor (Sanmin)	medicinal
Sedum hispanicum (Sedhis)	
Silene nutans subsp. dubia (Silnut)	
Stachys alpina (Staalp)	
Tragopogon orientalis (Traori)	
Trifolium aureum (Triaur)	
Trifolium ochroleucon (Trioch)	
Trifolium repens (Trirep)	
Verbascum nigrum (Vernig)	
Vicia cracca (Viccra)	
Viola tricolor (Viotri)	
