# Supplementary material for: A review of European studies on pollination networks and pollen limitation, and a case study designed to fill in a gap
Source: AoB Plants. 2018 Oct 31;10(6):ply068. doi: 10.1093/aobpla/ply068 (PMC6302952; doi:10.1093/aobpla/ply068)
Supplement: Supplementary Information S3 [file ply068_suppl_supplementary_information_s3.pdf]

Supporting Information Table S3: A list of all butterfly species observed in the meadow. Family, genus and species are recorded with some species being indistinguishable from each other grouped together and are listed in alphabetical order by genus. Species were appointed abundance categories based on the number of individuals observed in the meadow. 1-2 individuals were recorded as very rare, 3-9 rare, 10-50 common, and 50-1000 very common.

Genus	Species	Family	Abundance category
Adscita/Jordanita		Zygaenidae	Common
Aphantopus	*hyperantus*	Nymphalidae	Very Common
Argynnis	*adippe*	Nymphalidae	Rare
Argynnis	*aglaja*	Nymphalidae	Very Rare
Argynnis	*niobe*	Nymphalidae	Very Common
Argynnis	*paphia*	Nymphalidae	Very Rare
Autographa	*bractea*	Noctuidae	Very Rare
Boloria	*euphrosyne*	Nymphalidae	Rare
Brentis	*ino*	Nymphalidae	Very Rare
Coenonympha	*glycerion*	Satyridae	Very Common
Coenonympha	*pamphilus*	Satyridae	Rare
Cupido	*minimus*	Lyceaidae	Common
Erebia	*euryale*	Nymphalidae	Very Rare
Erebia	*ligea*	Nymphalidae	Rare
Erebia	*medusa*	Nymphalidae	Common
Gonepteryx	*rhamni*	Pieridae	Very Rare
Issoria	*lathonia*	Nymphalidae	Very Rare
Leiptidea	*reali/juvernica*	Pieridae	Very Rare
Limenitis	*camilla*	Nymphalidae	Rare
Lycaena	*alciphron*	Lycaeidae	Common
Lycaena	*virgauerae*	Lycaeidae	Very Common
Maculinea	*alcon*	Lycaeidae	Common
Maniola	*jurtina*	Satyridae	Very Common
Melanargia	*galathea*	Satyridae	Very Common
Melitaea	*athalia/britomartis/aurelia/parthenoides*	Nymphalidae	Common
Neptis	*rivularis*	Nymphalidae	Very Rare
Nymphalis	*io*	Nymphalidae	Rare
Nymphalis	*urticae*	Nymphalidae	Very Rare
Ochlodes	*sylvanus*	Hesperiidae	Rare
Polyommatus	*dorylas*	Nymphalidae	Rare
Polyommatus	*icarus*	Nymphalidae	Common
Pyrgus	*alveus/armoricanus*	Hesperiidae	Very Rare
Satyrium	*w-album*	Lycaeidae	Very Rare
Scotopteryx	*chenopodiata*	Geometridae	Very Rare
Thymelicus	*sylvestris*	Hesperiidae	Rare

Zygaena	*filipendulae*	Zygaenidae	Common
Zygaena	*lonicera*	Zygaenidae	Very Rare
Zygaena	*purpuralis/minos*	Zygaenidae	Very Common
